# Framing Attention in Japanese and American Comics: Cross-Cultural Differences in Attentional Structure

**DOI:** 10.3389/fpsyg.2012.00349

**Published:** 2012-09-24

**Authors:** Neil Cohn, Amaro Taylor-Weiner, Suzanne Grossman

**Affiliations:** ^1^Department of Psychology, Tufts UniversityMedford, MA, USA

**Keywords:** cultural psychology, visual language, comics, attention, manga

## Abstract

Research on visual attention has shown that Americans tend to focus more on focal objects of a scene while Asians attend to the surrounding environment. The panels of comic books – the narrative frames in sequential images – highlight aspects of a scene comparably to how attention becomes focused on parts of a spatial array. Thus, we compared panels from American and Japanese comics to explore cross-cultural cognition beyond behavioral experimentation by looking at the expressive mediums produced by individuals from these cultures. This study compared the panels of two genres of American comics (Independent and Mainstream comics) with mainstream Japanese “manga” to examine how different cultures and genres direct attention through the framing of figures and scenes in comic panels. Both genres of American comics focused on whole scenes as much as individual characters, while Japanese manga individuated characters and parts of scenes. We argue that this framing of space from American and Japanese comic books simulate a viewer’s integration of a visual scene, and is consistent with the research showing cross-cultural differences in the direction of attention.

## Introduction

Cross-cultural research shows that Asians and Americans differ in their direction of attention (Nisbett, 2003; Nisbett and Miyamoto, 2005). Beyond studying attention through perception, cognition can also be compared through cultural production (Morling and Lamoreaux, 2008), as in artistic expression (Masuda et al., 2008). Comic books provide an ideal place to analyze the direction of attention, because panels act like windows onto a scene (Cohn, 2007). Thus, analysis of panels in Asian and American comics provides a place to look for cultural differences in cognition through creative expression.

Cross-cultural differences in attention have been consistent across numerous behavioral paradigms. After viewing video scenes, Americans mostly describe the salient objects, while Asians describe significantly more aspects of the surrounding context (Masuda and Nisbett, 2001). Americans also tend to notice changes to focal objects in animations that feature slight changes to a single scene, while Asians pick up on changes to the broader environment and relations between objects (Masuda and Nisbett, 2006). When recalling scenes where the background is changed from its original context, Americans are unaffected while Asians’ memory appears impaired (Masuda and Nisbett, 2001), and Americans’ eye movements fixate sooner and longer on focal objects, while Asians make more saccades to elements of the background (Chua et al., 2005). Additionally, when viewing photographs of objects, fMRI studies show that Americans have stronger activation than Asians in brain regions associated with the storing of semantic information about object properties (Gutchess et al., 2006). All of this work supports that Americans focus more on focal objects while Asians attend more to aspects of environments and relationships.

Research has also suggested that preferences for attention permeate into artistic representations. Masuda et al. (2008) looked at a corpus of artwork, and found that “Western” paintings emphasized the focal objects and figures, while Asian paintings emphasized the broader context and environment. This trend was reinforced in drawings and photographs of figures and scenes produced by individuals from these cultures. Thus, these cognitive preferences for attention extend into artistic expression, and other contemporary media produced by these cultures might be expected to show further evidence of these trends.

Comic books are an ideal place to examine the focus of attention in artistic expression. Because comic panels act as a window on a visual story, they can simulate a “spotlight of attention” for a reader’s perception of a fictitious scene (a similar argument for film shots is made by Levin and Simons, 2000). Importantly, unlike the isolated images of photos and drawings, comic panels are meant to be read (and are created) in a sequence. Individual photos often include the whole field of vision of a single scene, and attention can be directed to parts of this scene in different ways. In contrast, sequential images serve as a window on unfolding events, with panels potentially simulating the way that attention might be directed on a scene. Indeed, recognition that the panel serves as a window appears to require some degree of exposure and practice: children’s drawings from Japan (where comics and visual representations are highly prevalent) use this windowing quality of panels to occlude parts of images far more often than those from Egypt, which has less prevalent cultural pictorial representation (Wilson and Wilson, 1987).

**Figure 1:**
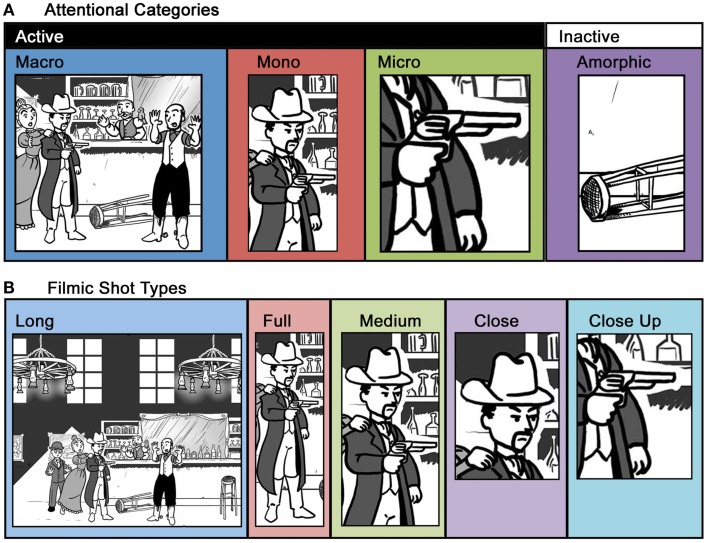
**A single scene framed by attentional categories (A) and filmic shot type (B)**. Attentional categories outline the meaningful elements of a scene, while filmic shot types present those meaningful elements in different ways.

With this view in mind, Cohn (2007) described comic panels as “attention units” that highlight parts of a scene in different ways. Within a sequence of images, a scene may have two types of meaningful elements: *Active entities* are those that repeat across panels by engaging in the actions and events of the sequence, while *inactive entities* are elements of the background. Panels can be categorized related to the ways that they depict these meaningful elements (see Figure 1A):

1. *Macro* – depict multiple active entities
2. *Mono* – depict single active entities
3. *Micro* – depict less than one active entity (as in a close up)
4. *Amorphic* – depict no active entities (i.e., only inactive entities)

These categories are distinguished by the amount of information they contain, which decreases successively: Macros contain more active information than Monos, which show more than Micros, which are more than Amorphic panels. These ways of highlighting attention are similar to types of film shots, though ultimately they differ in important ways. Thus, it is worth addressing these differences.

Film tradition has developed various conventional ways to frame figures and scenes based on what is being shown (Arijon, 1976; Bordwell and Thompson, 1997; Brown, 2002). There are many variations on the ways to frame figures and scenes; however, the main categories can be broadly defined as:

1. Long shot – figures are prominent in the frame, but the background dominates
2. Full shot – frames all of an entity or object (for example, a whole person or a whole car)
3. Medium shot – frames less than a whole entity, object, or scene (for example, when depicting a single person, Medium shots show the body from the knees or waist up)
4. Close shot – frames slightly more than a particular part of an entity or object, though less than a Medium shot (as in a person’s torso and up)
5. Close up – zoom in on a particular part of an entity or object (as in a person’s head or closer)

These divisions create framing for various aspects of scenes and people, as depicted in Figure 1B. Unlike the attentional categories, filmic shots frame the presentation of objects, as opposed to dividing the amount of information shown. In essence, attentional categories outline the framing of *meaningful* elements of a scene, while film shots describe the *presentation* of those meaningful elements. For example, a Mono panel shows only one character, as in the Gunman in Figure 1A. However, that character can be presented in various ways, including Full, Medium, and Close shots, as in Figure 1B. These are all ways in which to present the same meaning.

Nevertheless, the overlap between attentional categories and film shots should immediately be apparent, and prototypical correspondences may exist between them. For example, a Macro may typically involve a Long shot to capture the most information possible, as in the Long shot in Figure 1B, but it could tighten on just the specific multiple characters involved in the action, like the Macro in Figure 1A. This would be a Medium shot. Also, a panel showing only the hands of individuals exchanging a piece of paper would be a Macro that uses a Close up shot, because it involves multiple characters. Along these lines, Close ups may prototypically be Micros, but this varies based on how much information they window. Similarly, Amorphics have no equivalent category in film shots, since they show a non-active element of the narrative, which can be framed in any number of ways.

With the growing influx of Japanese manga (“comics”) into the United States over the past several decades (Goldberg, 2010; Wong, 2010), much comparison has been made between the techniques of Japanese and American authors (McCloud, 1993, 1996; Rommens, 2000; Cohn, 2010, 2011). Japanese manga come from a different cultural context than that of American comics. While comics in the USA have historically appealed to a particular subculture, manga in Japan are treated much the same as movies, television, or textual books. Manga are widely read by all ages, have many genres, and, in fact, are so popular that they constitute nearly one-third of all printed material (Schodt, 1983, 1996; Gravett, 2004). Though Japanese manga were influenced by American authors early in their historical development (Gravett, 2004), they developed largely in isolation over the past 60 years. With increased importation of manga into America starting in the 1980s, the differences between narrative techniques that emerged from these separate traditions have become quite salient to readers, authors, and scholars of comics in America.

In one of the first comparisons of American and Japanese comics, McCloud (1993) coded the semantic relationships between juxtaposed panels. He found that American authors primarily used transitions showing actions with clear temporal change, followed by shifts between characters (one character to another) and scenes (as in a shift from one whole spatial location to another). Manga similarly showed shifts in actions, characters, and scenes. However, unlike American books, manga also transitioned to different aspects within a scene, such as using panels to solely depict parts of the surrounding environment (i.e., Amorphic panels). The Amorphic panels give the sense of a “wandering eye” across the scene, and were introduced into manga from the influence of Japanese cinema in the 1950s (Shamoon, 2011). McCloud attributed the differences between cultures’ panels to an “artistic culture” of Japan that focused on “being there over getting there.” However, these findings are similar to the attention research: American comics focus on actions and figures while Japanese manga also include information about the surrounding environment.

**Figure 2:**
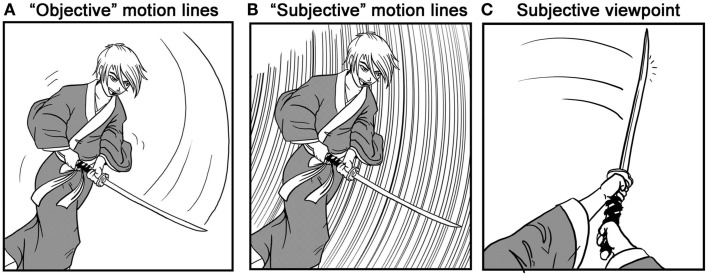
**“Objective” motion lines (A) use a line that is symbolic of the path an object travels, while “subjective” motion lines (B) “blur” the background as if the viewer were moving at the same pace as the object**. In subjective viewpoints **(C)** the panel is shown through the perspective of a character in the narrative.

McCloud (1993, 1996) has also proposed that manga allow a reader to take more of a “subjective” viewpoint on a story than American comics – meaning that manga use techniques that immerse the reader in the narrative as if it were perceived through their own viewpoint, instead of an omniscient “objective” perspective. Such a distinction is especially important if panels are thought to be units of attention, since that framing would then take on the “subjective” point of view of a reader that might differ between cultures. McCloud based his cross-cultural comparison on several factors including the greater focus on environmental aspects in storytelling, which reflect a “wandering eye” across the scene. Second, manga use more “subjective” types of motion lines where a reader appears to move at the same pace as a moving object, thereby seeing the object as solid while the background is blurred (Figure 2B), as opposed to seeing it move in front of them, where the path itself becomes a blur (as a motion line), as is found in American comics (Figure 2A). Finally, manga were said to use more subjective viewpoints in panels, which show the viewpoint of a character in the narrative (Figure 2C). In order to test this broad claim directly, Cohn (2011) coded a corpus of comics and manga for this last type of subjectivity, where panels depict the viewpoint of a character in the narrative. More subjective panels were used in Japanese manga than American comics. This provided evidence that manga do indeed use more subjective viewpoints, at least across one measurable dimension.

Cohn’s (2011) study also examined the attentional types of panels described above. Nearly 60% of American panels were Macros, with only 35% Monos and 5% Micros (Amorphics were not yet theorized as a category, and were likely mixed in Monos and Micros). However, Japanese manga used almost as many Macros (47%) as Monos (43%), and more Micros (10%) than American comics. Because manga featured less than the whole scene in over half of all panels, it implies that the Japanese are as interested in the component parts of a scene as much as the whole scene. These results also suggest that the narrative structure of manga demands the inferential construction of whole scenes more than American comics (Cohn, 2010). These findings of more Micros in Japanese manga are also consistent with claims by Toku (2001, 2002) that manga influences Japanese children’s drawings. She found that Japanese children draw far more variable viewpoints than American children, particularly “exaggerated” close ups.

**Figure 3:**
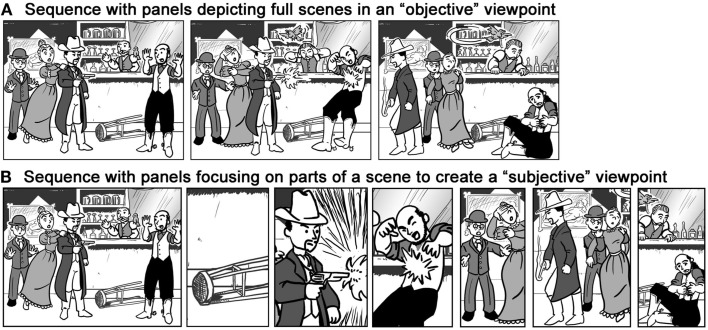
**A sequence with an “objective” viewpoint (A) depicts all the components from a scene, letting the viewer pick out the important parts**. A “subjective” viewpoint **(B)** lets each panel frame only the important parts, thereby simulating the spotlight of attention on a scene.

These studies suggest a difference overall between panels in American comics and Japanese manga that could be construed as reflecting the differences in cross-cultural windowing of attention. Like in attention, readers track only the most important aspects of a sequence to establish the continuity of the narrative. Non-relevant information may then go unattended by the “spotlight of attention” across panels, as happens in change blindness paradigms (Levin and Simons, 2000). There are thus two strategies comic authors can use when creating a comic. One option is to show a full scene (Macro) and rely on the reader’s attentional intuitions to discern the most important parts. In this “objective” method, a reader’s personal spotlight of attention selects the relevant information in a scene (Figure 3A). Alternatively, authors can use panels to highlight salient parts directly, omitting what is unimportant altogether. This use of panels would heighten panels’ ability to depict a “subjective viewpoint,” since the panels would *become* the spotlight of attention to focus only on important parts of a scene (Figure 3B).

The previous research suggests that American comics more consistently use the first option: authors provide an objective viewpoint on a scene, letting the reader direct their own attention across panels to find the most relevant aspects of continuity, while less important elements simply go unattended. This is suggested by the larger amounts of Macros found in American comics. In contrast, Japanese manga do more to simulate the perception of a reader’s attention, evident in greater use of Monos and Micros. This “subjective” strategy of Japanese manga is consistent with McCloud’s (1993, 1996) claim that manga allow a reader to take more of a subjective viewpoint on a story. It also is supported by previous corpus analysis showing that “subjective viewpoints” are more plentiful in Japanese manga than American comics (Cohn, 2011). Thus, rather than Japanese panels showing large scenes (Macros) that include aspects of the background (as in the study of art and photographs by Masuda et al., 2008), panels in manga *directly* depict these elements of a scene (Monos, Micros) because of the way that panels simulate the window of attention.

Thus, this previous research could support that comic panels serve to simulate attention on a fictitious scene in ways that are consistent with cross-cultural differences in attention. However, an alternative possibility is that these differences merely arise because of separate narrative conventions between American and Japanese comic authors. Indeed, while these studies have shown that comic panels vary between cultures, panels may also differ within cultures. Obvious variability can be found in the diversity of American graphic styles compared to the far more uniform drawing style in manga. Graphic styles are particularly pronounced between genres, such as between the more “serious” Independent graphic novels, which range from more straightforward and realistic styles to cartoony styles, and mainstream comics, which have the bombastic style of muscular heroic figures (Duncan and Smith, 2009). Styles in genres of Japanese comics also vary (Schodt, 1983, 1996; Gravett, 2004), but primarily conform to the stereotypical style of big eyes, pointy chins and noses, and big hair. The diverse styles used in American comics have been likened to types of “dialects,” compared with “accents” in manga genres, which feature variations on a common schema (Cohn, 2010).

Some research suggests that variation between genres extends to the level of panels, and can thereby inform about the framing of attention. In an early study, Neff (1977) found that panels from various genres of American comics use film shots differently. Wide shots (Long and Medium) far outnumbered Close shots (Close and Close ups) in panels for all genres. However, there were far fewer Close shots in Adventure and Romance comic panels than in Mystery and Alien Beings comics. These findings imply that various genres of American books do highlight diverse aspects of a visual scene. However, the sample size in this study was somewhat limited in scope – only two pamphlet-sized comics were analyzed per genre – making the results hard to generalize.

If differences between panels in American comics and Japanese manga are a reflection of cross-cultural differences in attention, these trends should transcend differences between genres within those cultures. Indeed, Cohn’s (2011) study mixed together various genres within the overall samples of American comics and Japanese manga. Thus, the present study sought to examine comic panels both within and between cultures by comparing the panels of “mainstream” Japanese manga with the two major genres of American comics: Mainstream and Independent (“Indy”) books. Mainstream books from both the United States and Japan were chosen because they are the most popular and most stereotypical instances of their respective comic cultures. American Indy books were chosen because they presented a different artistic movement in the USA that contrasts the Mainstream genre (discussed below). Thus, if variation occurs between the structures of comics from within the United States, we may expect it between Mainstream and Indy comics.

If the differences between American comics and Japanese manga are merely an artifact of different narrative conventions, we would expect that Indy books would also have their own unique conventions of storytelling that define them from Mainstream American comics. Under this view, we predicted that attentional categories from all three groups would differ. Because no previous studies have yet examined Indy comics in this way, it is difficult to predict how trends of attentional categories may differ from Mainstream comics.

Nevertheless, if American genres show no variation, yet are both different from Japanese manga, it would imply cultural differences beyond the contexts of genre. To this end, we would predict the results to replicate the study by Cohn (2011) that showed a greater focus on whole scenes (Macros) by panels in American comics, and a greater focus on parts of scenes (Monos, Micros) by Japanese manga. Furthermore, consistent with McCloud’s (1993) findings that manga focus more on surrounding aspects of a scene, we would expect panels from Japanese manga to use more Amorphic panels than American comics. Such results would provide additional support that the attentional windowing in panels from comics reflect broader trends in cross-cultural cognition in attention.

## Materials and Methods

### Materials

Thirty graphic books were chosen at random from a corpus of over 200 comics donated from various comic companies. Companies were told only that these works would be used for research on the cognition of comics. Ten books were chosen from each of three groups: “mainstream” Japanese Manga, Mainstream American comics, and Indy American comics. In order to operationalize how these groups are identified as belonging to different narrative traditions, it is useful to discuss their differences.

Mainstream and Indy books differ greatly in graphic styles, genres, formats, publishers, and often readership. Mainstream comics primarily feature drawing styles common to superhero comics (dynamic line work, muscular figures, brighter colors), and focus on the genres of superheroes, horror, and science fiction. Mainstream books also are often produced by specific publishers and are serialized in pamphlet style formats that are only sometimes afterward collected into books. Mainstream comics are sold primarily through specialty comic books stores. In contrast, Indy books use more variable graphic styles (particularly more cartoony and “artistic” styles) with more “serious” or dramatic genres (such as memoir, drama, etc.). Different publishers are known for producing Indy books and Mainstream comics, and they appear mostly in book formats (“graphic novels”). Indy books are often sold in comic books stores, but also have a much higher distribution into regular bookstores.

While some overlap in readership does exist between Mainstream and Indy comics, they largely appeal to different groups of people. Readers of Mainstream comics often read serializations that appear each month. They often are very devoted to their favorite comics, and American comics often target the writing with this consistent readership in mind, evident through frequent references to previous storylines. Indy comics have more varied readership because they are not serialized volumes. Often, Indy books are produced in single editions, and thus do not have consistent readership (though readers may follow particular authors’ works). Readers of Japanese manga are often more similar to Mainstream American comics – they have their favorite comics which are released weekly in large anthologies. While readership of manga is larger on the whole in Japan than America, there is no reason to believe that comics in either country are explicitly made with any expectation that readers will be more or less proficient in understanding them.

Additionally, while some crossover exists in readership between American genres, most authors of Mainstream and Indy books remain independent of their genres. Mainstream and Indy books are also created with a slightly different process. Mainstream comics are largely made by an industry-line style committee (Duncan and Smith, 2009) consisting of a writer, penciler, inker, colorist, etc. While an editor coordinates their efforts and oversees the plotline, for the most part these creators are free to follow their own styles of writing and artwork. In contrast, Indy comics are more often drawn and written by individual authors who fulfill all of these functions themselves. Japanese manga typically combine these methods. They are usually attributed to a sole author, who then employs a team of uncredited assistants who complete the more menial aspects of the drawings, like shading or drawing backgrounds (Schodt, 1983).

In this study, we distinguished American Mainstream and Indy books by criteria of graphic style, genre, and publishers. Mainstream books ranged in publication date from 1992 to 2005 with a mean of 2002, while Indy comics were published between 1991 and 2008, with a mean at 2003. Japanese books featured more consistent visual styles, following the stereotypical “standard graphic dialect” of Japanese comics (Cohn, 2010). However, since genres in Japan do not align neatly with those in America (Shonen “boys comics,” Shojo “girls comics,” and Gekiga “serious comics”), books were chosen that reflected the genre closest to Mainstream American comics – those focusing primarily on action/adventure themes (Shonen “boys comics”). Only English translations of manga were analyzed in the study due to their availability in our donated corpus, though manga were attributed to their original Japanese publication dates, from 1984 to 2005 with a mean of 1999.

Thus, our analysis contrasted both narrative tradition and country of origin. American Mainstream books shared a similar overall genre (action/adventure) with Japanese manga, though they came from the same country of origin as American Indy comics. All of the chosen books were widely read and popularly distributed throughout comic readership, and from major publishers – i.e., none of the books were obscure or minimally distributed. A full listing of books analyzed is provided in the Appendix.

### Areas of analysis

All books were coded across two primary dimensions: Attentional Category and Shots. Attentional Category coded the way in which panels highlight attention in the various types of attentional categories previously discussed (Macro, Mono, Micro, Amorphic). Panels that could not be identifiably coded into these categories were recorded as “Ambiguous.” Additionally, panels were coded for the type of filmic shot used in a panel (Long, Full, Medium, Close, Close up).

We coded 300 panels (or the closest number therein to the nearest completed page) in each book, and found the mean number of coding per book per coder. Samples were fairly similar in the number of pages/book and panels/page analyzed. Indy comics averaged 56.6 pages/book and 5.99 panels/page, while Mainstream comics averaged 62.6 pages with 5.12 panels/page. Manga used 65.2 pages/book with 4.75 panels/page.

Two researchers blind to the hypothesis of the study independently coded each book for both attentional category and shot type. Coders were found to be generally consistent, and we found an inter rater reliability for attentional categories to be Kappa = 0.785, *p* < 0.01, and for shots to be Kappa = 0.541, *p* < 0.01. The mean attentional categories or shots were found for each book by each coder, and final analyses used the mean between coders’ scores for each book.

### Statistical analysis

Attentional categories and shot types were analyzed across samples using Mixed Model ANOVAs that set “Group” (i.e., American Mainstream, American Indy, and Japanese Manga) as the between-subjects factor and either Attentional Category (i.e., Macro, Mono, Micro, Amorphic) or Shots (i.e., Long, Full, Medium, Close, Close up) as the within-subjects factor. Follow up ANOVAs and *t*-tests looked at the differences of each attentional categories and shot type within and between groups.

## Results

### Attentional category

The analysis of attentional framing of panels found a main effect for Attentional Category, *F*(3,81) = 89.71, *p* < 0.001, with an Attentional Category by Group interaction, *F*(6,81) = 5.68, *p* < 0.001. A main effect between Groups was not significant, *F*(2,27) = 1.37, *p* = 0.269.

**Figure 4:**
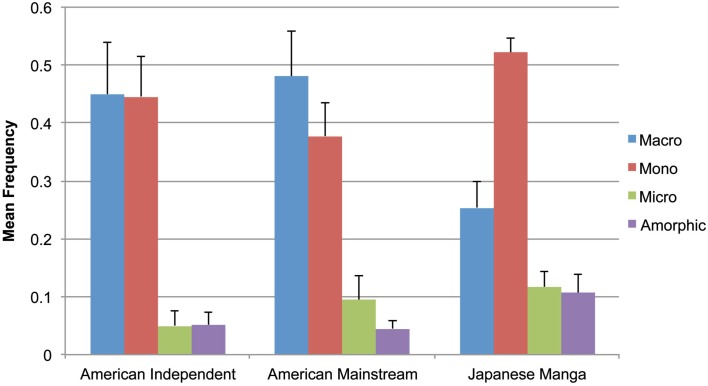
**Mean frequency of attentional categories in American Independent and Mainstream comics and Japanese manga**.

As depicted in Figure 4, both Indy and Mainstream comics used large amounts of Macros and Monos, with minimal Micros and Amorphics. Within Indy comics, a main effect was found between attentional categories, *F*(3,27) = 27.34, *p* < 0.001, and differences were also found between all pairs of types (all *t* > 5.81, all *p* < 0.001), except the near equal means for Macros with Monos, and Micros with Amorphics. We also found a main effect between categories of Mainstream panels, *F*(3,27) = 30.05, *p* < 0.001. These books featured only slightly more Macros than Monos, which was not statistically significant. Micros and Amorphics numbered far fewer overall, though there were almost twice as many Micros as Amorphics, a difference trending in significance, *t*(9) = 2.14, *p* = 0.06. All other attentional categories featured significant contrasts (all *t* > 5.55, all *p* < 0.001).

Finally, a main effect between attentional categories was found in Manga panels, *F*(3,27) = 64.00, *p* < 0.001. Here, Monos far outnumbered other types, with roughly half as many Macros, and far fewer Micros and Amorphics. All pairwise relationships between attentional categories featured significant differences (all *t* > 3.16 or <−7.3, all *p* < 0.05), except between Micros and Amorphics.

Comparison of the distribution of each Attentional category between all three groups showed either significant or trending relationships, as summarized in Table 1. Indy and Mainstream comics showed no differences for any of the attentional categories. Indy panels were significantly different from Manga for all types except Monos, while Mainstream panels differed from Manga except with regard to Micros. Additionally, while not statistically significant, it is notable that standard deviations in Manga were far lower than both types of American comics in Macros and Monos.

**Table 1:** **Comparisons of attentional categories and shots between groups**.

	Omnibus	Indy versus mainstream	Indy versus manga	Mainstream versus manga
	*F*, *p*	*t*, *p*	*t*, *p*	*t*, *p*
**ATTENTIONAL CATEGORIES**				
*Macro*	7.04, 0.003*	−0.52, 0.61	3.74, 0.005*	4.05, 0.003*
*Mono*	4.30, 0.02*	1.19, 0.26	−1.71, 0.12	−2.91, 0.02*
*Micro*	2.81, 0.08	−1.77, 0.11	−2.996, 0.02*	−0.63, 0.54
*Amorphic*	5.22, 0.01*	0.62, 0.55	−2.66, 0.03*	−3.65, 0.005*
**FILM SHOTS**				
*Long*	1.19, 0.32	−0.36, 0.73	1.22, 0.25	2.2, 0.055
*Full*	1.32, 0.28	0.18, 0.86	1.35, 0.21	1.67, 0.13
*Medium*	6.74, 0.004*	2.61, 0.03*	3.70, 0.005*	0.79, 0.45
*Close*	6.12, 0.006*	−0.55, 0.60	−3.90, 0.004*	−3.26, 0.01*
*Close up*	3.63, 0.04*	−2.29, 0.048*	−3.33, 0.009*	−0.7, 0.50

Omnibus *N* = 30, pairwise relations *N* = 10, *p < 0.05.

### Shots

Analysis of all panels for their type of filmic shots showed a main effect for Shots, *F*(4,108) = 12.49, *p* < 0.001, as well as a Shot by Group interaction, *F*(8,108) = 3.72, *p* < 0.001. No main effect between Groups was found, *F*(2,27) = 1.40, *p* = 0.265.

**Figure 5:**
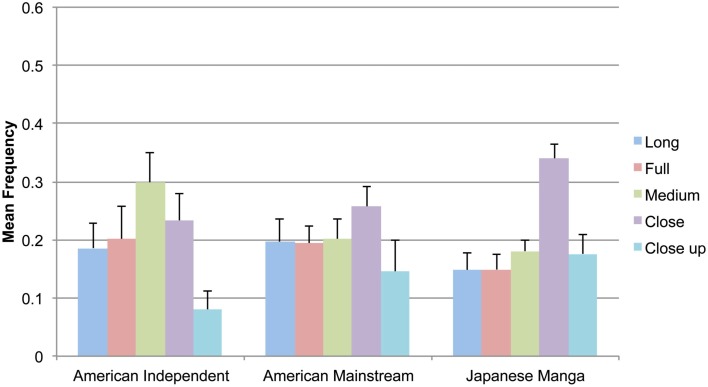
**Mean frequency of types of filmic shots in American Independent and Mainstream comics and Japanese manga**.

Means for shot types in all groups are depicted in Figure 5. American Indy comics used Medium shots the most, followed by Close shots, with minimal use of Close ups. A main effect of Shots was found across panels in Indy comics, *F*(4,36) = 6.18, *p* < 0.005. Significantly less Long shots were used than Medium, *t*(9) = −2.37, *p* < 0.05, and Close shots, *t*(9) = 2.87, *p* < 0.05. Close ups were also found to be used less than Full *t*(9) = 2.80, *p* < 0.05, and Medium shots, *t*(9) = 4.81, *p* < 0.005. Despite using slightly more Close shots and less Close ups than any other types, no main effect of Shots was found across Mainstream panels, *F*(4,36) = 2.04, *p* = 0.110. A main effect of Shots was also found in Manga panels, *F*(4,36) = 17.33, *p* < 0.001. Close shots were used significantly more than all other types (all *t* < −5.6 or >6.05, all *p* < 0.05). Pairwise relations between other shot types were not significant.

All three groups differed significantly only in their use of Medium, Close, and Close up shots. Pairwise comparisons showed that Indy comics used more Medium shots than both Mainstream comics and Manga, but fewer Close up shots. Manga used more Close shots than both American types. These statistics are summarized in Table 1.

### Attentional categories by shots

**Figure 6:**
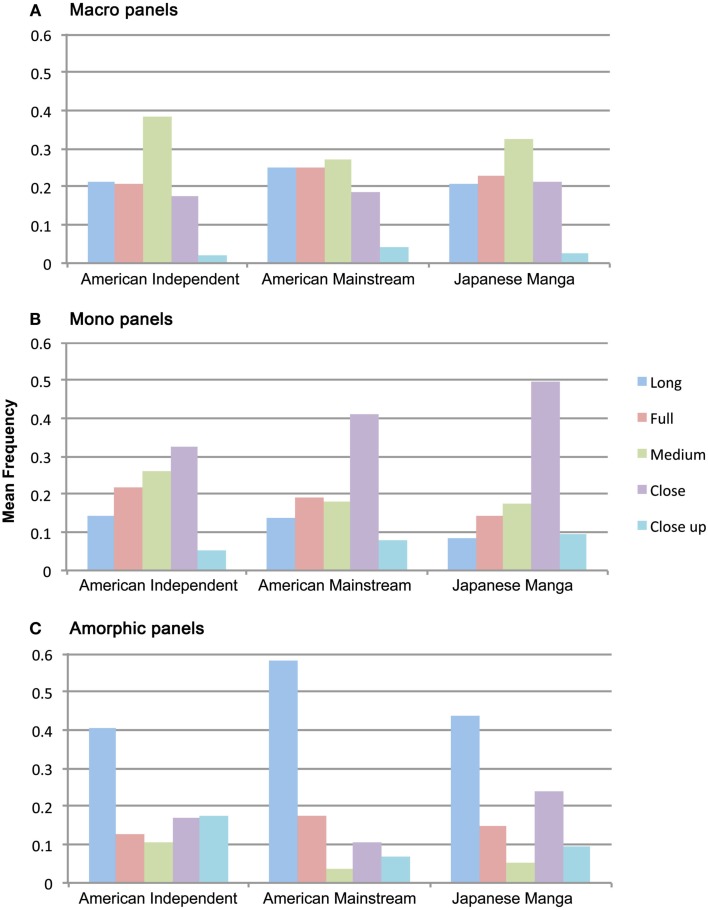
**Proportion of attentional categories–-(A) Macros, (B) Monos, (C) Amorphic panels–-represented by each of the filmic shot types in American Independent and Mainstream comics and Japanese manga**.

We followed our initial analysis of attentional categories and shots by looking at the relationship of these codings. Across all groups, Macro panels did differ in the proportion of shot types that they used, *F*(4,108) = 37.87, *p* < 0.001, however, no Shot × Group interaction was found, *F*(8,108) = 1.28, *p* = 0.260. As expected, Macro panels very rarely used Close up shots, but consistently used most all other shot types, with particular emphasis on Medium shots (see Figure 6).

Mono panels also differed across all groups in the proportion of shot types that they used, *F*(4,108) = 65.84, *p* < 0.001, and showed differences of shot types between groups, *F*(8,108) = 3.99, *p* < 0.001. As depicted in Figure 6, Close shots significantly outnumbered all other shot types in both Mainstream comics and Manga (all *t*s > 3.1, all *p*s < 0.05), where other shot types remained comparatively low. Mainstream panels also used less Close ups for Monos than all other shots except Long shots (all *t*s 3.1, all *p*s < 0.05). Similarly, Monos in Manga used Long and Close up shots significantly less than all other shot types (all *t*s > 2.3, all *p*s < 0.05). Close shots were also used the most prevalently in American Indy panels, but not significantly more than Full or Medium shots. These three shot types – prototypic of framing individuals – were used significantly more than Long or Close up shots (all *t* > 2.3, all *p* < 0.05). Between groups, more Long and Close shots were used in Indy Mono panels than Manga panels (all *t*s > 2.9, all *p*s < 0.05).

Micro panels further showed differences collapsed across groups for the proportion of shot types, *F*(4,108) = 737.8, *p* < 0.001, but no interaction between Shots and Groups, *F*(8,108) = 0.974, *p* = 0.460. In all three groups, Micro panels were dominated by Close Up shots (Indy comics: 87%, Mainstream: 95%, Manga: 96%). Other than Indy comics, where Close shots accounted for 7% of Micros, all other Micros were less than 4% of all other shot types in each group.

Finally, differences between shot types were found across all groups for Amorphic panels, *F*(4,108) = 34.57, *p* < 0.001, while a trending interaction between Shots and Groups was found, *F*(8,108) = 1.96, *p* = 0.059. In all groups, more Amorphic panels were presented as Long shots than any other shot type except as Close shots in Indy panels (all *t*s > 2.1, all *p*s < 0.064). These views likely showed the exterior of buildings and locations as a whole (as in establishing shots), as depicted in Figure 6. Between groups, Long shots showed only a trending difference between Mainstream and Manga panels, *t*(9) = 2.0, *p* = 0.077. Manga also used Close shots significantly more than all other shots except Long shots (all *t*s > 2.5, *p* < 0.05). Compared to other groups, Manga used more Close shots for Amorphic panels than Indy panels, which in turn were greater in number than Mainstream panels (all *t*s > 2.2, all *p*s < 0.05). These Close shot Amorphic panels perhaps reflect the “wandering eye” representations that show aspects of a surrounding environment said to be more prevalent in manga than American comics (McCloud, 1993; Shamoon, 2011).

## Discussion

This study analyzed how various cultures’ comic panels frame a fictitious scene as a way to gain insight on how these cultures may direct attention. We compared Mainstream and Indy genres of American comics with “mainstream” Japanese manga. Even more than in Cohn’s (2011) study, Japanese panels highlighted the component parts of scenes more than American books. Japanese manga were found to have far more Monos than any other type of panel, followed by Macros, and small proportions of Micros and Amorphics. Both Mainstream and Indy American comics had near equal proportions of Macros and Monos, again with small proportions of Micros and Amorphics.

In the analysis of attentional categories between cultures, manga used significantly more Monos, Amorphics, and Micros than did both types of American comics. American comics did not vary in their attentional categories between genres, despite surface stylistic differences. Thus, though Japanese manga and Mainstream American comics were similar in terms of “mainstream” appeal and action/adventure themes, this similarity did not influence the framing of scenes. These results suggest that the primary difference between these groups of comics are that of country of origin: The framing of entities in American comic panels differ from Japanese panels, though American comic genres do not differ substantially from each other.

Regarding film shots, we found that all three groups differed from each other. In this case, Indy comics differed the most from the other two groups, focusing highly on Medium shots with very few Close ups. In contrast, Japanese panels were dominated by Close shots, with near equal amounts of all other types. Mainstream comics showed the same overall pattern as manga, though with far fewer Close shots. These findings indicate the opposite of the study of attentional categories: the choice of filmic shots differs more based on genre than country of origin. Mainstream American books were similar to Japanese manga, sharing the overall genre of “action/adventure,” while Indy comics were different from them both.

Analysis of the proportion of shot types used by each attentional category further illuminated the ways that content (attentional categories) were presented (shots). Some prototypical correspondences emerged quite strongly, such as Micro panels being dominated by Close ups, and Amorphic panels dominated by Long shots (likely for exterior locations) and in Manga, Close shots (for focusing on elements within an environment). However, contrary to the idea that full scenes in Macros would be shown by the widest viewpoint (Long shot), they often used Medium shots. This may reflect that panels may not show whole bodies in character interactions, though they must have enough space to depict multiple characters. Similarly, Mono panels were largely framed using Close shots – a tight representation of an individual. These results for Macros and Monos likely reflect an optimization of depicting one or multiple characters in the limited physical space of a panel on a page.

Nevertheless, the proportion of shot types used by each attentional category differed only minimally between groups, and only for slight variances in using Mono and Amorphic panels. This widespread similarity suggested that the authors of each of these types of books by and large use the same presentation (shot type) of meaning (attentional categories). However, the variation in attentional categories between groups reflects that American and Japanese authors make different choices for what content should be highlighted throughout a narrative sequence.

What can these results offer to our understanding of cross-cultural attention and cognition? The framing of attention in both genres of American comics depicted a whole scene as much if not more than individual characters, as indicated by Macros having equivalent (Indy) or greater (Mainstream) prevalence than Monos. In contrast, Japanese manga directed attention toward details in the scene through Monos, Micros, and Amorphics, in lieu of actually showing full scenes in Macros. These results are consistent with Cohn’s (2011) study that showed a greater focus on whole scenes (Macros) by panels in American comics, and a greater focus on parts of scenes (Monos, Micros) by Japanese manga, as well as McCloud’s (1993) analysis showing more viewpoints within an environment (Amorphic panels) in Japanese manga.

We argue that these data support that attentional categories differ between cultures in ways predicted by differences in cross-cultural cognition. As discussed, attention can be directed throughout a sequence of images in two ways. First, an author can show a full scene (Macros) and rely on the reader to appropriately cast their spotlight of attention on the relevant parts. Alternatively, authors can use panels as the spotlight of attention to focus only on important parts of a scene. We have hypothesized that the more “subjective” nature of Japanese manga panels reflects authors using their panels to take this second strategy. Here, Monos, Micros, and Amorphic panels are used to highlight the component parts of a scene or environment because that would be how readers’ attention intuitively perceives elements of a visual array. However, out of this information readers need to inferentially integrate these parts into a coherent whole. In contrast, American authors are less concerned with providing this subjective viewpoint, and thus can use more Macro panels to show the entirety of a scene. Consequently, American readers will naturally pick out the focal characters of the scene, directing their own spotlight of attention to the important elements of interest automatically. In this way, panels from comics and manga may reflect cross-cultural differences in visual attention.

A crucial distinction in this interpretation is the “subjective” viewpoint taken by Japanese authors that is not used (or used less often) by American authors. We might expect that, without such an overarching principle, Japanese manga would use more Macros to uphold the attentional focus on environmental parts of a scene instead of just figural objects. However, because of this subjectivity, authors of Japanese manga are more apt to use panels as a framing device that simulates the perspective of a person’s eye on a scene, thereby honing in on its component parts. It is important to note that this type of storytelling introduces more complexity to the narrative grammar and demands more inference from readers (Cohn, 2010, in press), and indeed average Japanese university students (not necessarily comic fans) have shown higher aptitude for assimilating sequential images in a battery of “comics comprehension tests” than American students (Nakazawa and Shwalb, 2012).

Nevertheless, even with the more subjective viewpoint, the focus on background environment could still be more prevalent in Japanese manga panels than in American comic panels. Despite the stereotypically cartoony figures, manga panels often feature elaborately detailed backgrounds (McCloud, 1993). It would be interesting to know whether the direct representations of environmental versus figural objects shown in artwork by Masuda et al. (2008) correlates with this type of sequential representation where parts of a scene can become individuated by a whole panel. Thus, future work could not only look at the framing of attentional content, but could also code each panel for (1) the presence of background information, (2) the relative space allotted to backgrounds versus figures, and (3) detail given to backgrounds versus figures. We may predict, for example, that even though manga panels would individuate the component parts of scenes more often (due to a subjective viewpoint), background information in those panels would be more detailed and occupy more relative space in a frame than in American comic panels.

One concern for this study is that American books over the past two decades have already become greatly influenced by Japanese manga (Horn, 1996; McCloud, 1996; Goldberg, 2010; Wong, 2010). While the offset in dates between books in our corpus is marginal for their comparison between each other, these dates (1990–2000) do reflect an era of American comics that saw a massive increase in influence by Japanese manga. It may be the case that this particular sampling of American books already reflects this Japanese influence, and that a comparison of older works would yield more widely disparate trends in attentional categories. Such an interpretation provides clues as to why these data may differ from those of Cohn’s (2011) study. Here, we saw the same overall trends that Japanese panels focus on less than the whole scene more than American panels. However, American books had twice as many Macros as Monos, while the present results found nearly equal amounts of these types. Given that this original study used works by several older artists, this change could be attributed to American authors being influenced by manga in more recent years. However, future longitudinal research would be required to support this interpretation.

If American authors have indeed been influenced by Japanese manga to change their use of attentional categories, it calls into question how much these framing patterns reflect cognitive trends and how much they are shaped by the conventions of the visual narrative system. To answer such a question, it may be useful to study samples of visual narrative from Asia and America that are explicitly imitative of manga. For example, “manhwa” has most of the same style and conventions as manga, only they are produced in Korea (Lent, 2010). In America, “Original English Language manga” (OEL manga) fully imitate the graphic styles and conventions of Japanese manga, and are produced by Americans (Cha and Reid, 2005; Brenner, 2007). If attentional categories are artifacts of culture alone, we might expect manhwa to resemble the trends of Japanese manga, while OEL manga would pattern like American comics, despite their graphic style. However, if the trends of OEL manga resemble those of Japanese manga, it would provide evidence that the conventions of the visual narratives transcend their cultural contexts. Such findings would also call into question the degree to which these attentional categories actually can *shape* the attentional preference of their authors and readers. Would American readers and creators of manga have attentional preferences closer to those of Japanese?

Finally, additional follow up studies could focus on the differences between individual drawings and those intended for narrative in comics. The hypothesis here is that panels in comics simulate the attentional preferences of Japanese and Americans for understanding events across a sequence of images. Thus, if similar events were represented simultaneously in a large single image, we would expect to find attention to be directed similarly to how panels frame their content: Americans would be expected to fixate more on main characters and Japanese fixations would include peripheral information. Such a study would provide a useful bridge between previous studies of individual images from these cultures (Nisbett and Masuda, 2003; Masuda et al., 2008) and these studies analyzing sequential images that convey events.

By analyzing comics in this way, we have shown that visual narratives are bound by cultural conventions that create patterns in the ways that Japanese and American comic authors window attention onto visual scenes. We propose that these results are consistent with the cross-cultural research showing differences in how Asians and Americans perceive and attend to their visual environment, and further support efforts to study cognitive process through creative cultural expression.

## Conflict of Interest Statement

The authors declare that the research was conducted in the absence of any commercial or financial relationships that could be construed as a potential conflict of interest.
